# Is soluble transferrin receptor a good marker of iron deficiency anemia in chronic kidney disease patients?

**DOI:** 10.4103/0971-4065.57105

**Published:** 2009-07

**Authors:** S. Gupta, B. Uppal, B. Pawar

**Affiliations:** Department of Biochemistry, CMC Ludhiana, Punjab, India; 1 Department of Nephrology, CMC Ludhiana, Punjab, India

**Keywords:** Chronic kidney disease, erythropoietin, hemodialysis, iron deficiency anemia, soluble transferrin receptor, transferrin saturation, total iron binding capacity

## Abstract

Anemia in patients with chronic renal failure is multifactorial with an absolute or functional iron deficiency present in 60–80% of patients. In this study, 102 patients of stage 5 chronic kidney disease (CKD) were enrolled. Thirty six age- and sex-matched anemic patients without any known renal disease were taken as controls. Their sTfR levels were measured with anemia profile.(Fe, TIBC, Ferritin, TSAT). The patients were followed up twice, at four weeks and six months. There was a significant statistical difference in the mean sTfR levels in patients when compared to controls (*P* < 0.01).The mean level of sTfR in CKD patients was 3.23 ± 2.07 mg/l while in controls this was 5.16 ± 3.64 mg/l. sTfR had no statistically significant correlation with the levels of hemoglobin, iron, ferritin, TIBC and TSAT. We conclude that owing to complexity of iron metabolism in CKD, sTfR can not be used as a reliable marker of iron deficiency anemia.

## Introduction

Chronic kidney disease (CKD) is an important, chronic, noncommunicable epidemic disease that affects the world, including India.[1] Anemia affects 60–80% of patients with renal impairment, reduces the quality of life and is an additional risk factor for early death. Anemia of CKD, especially in patients on hemodialysis, is of multifactorial etiology. Absolute or functional iron deficiency is present in 25–38% of patients with anemia of CKD.[2] Serum ferritin and transferrin saturation (TSAT) are the most commonly done tests used for diagnosing iron deficiency anemia (IDA). However, these standard tests do not consistently reflect the iron status of patients with CRF on hemodialysis.[3]

In recent years, soluble transferrin receptor (sTfR) has been introduced as a sensitive, early and highly quantitative new marker of iron depletion, increasing in proportion to tissue iron deficit.[4] The transferrin receptor (TfR) is a homodimeric type II membrane protein that mediates iron uptake into the cells.[5] As the TfR-transferrin-iron complex reaches the cell membrane it is internalized via an endocytic vesicle. In the intracellular compartment, iron dissociates from TfR-transferrin complex. The iron remains in the cytosol while the TfR-transferrin complex is recycled back to the cell surface.[6] During the process of recycling, some of these TfR are shed in to the blood and appears as soluble or serum transferrin receptor (sTfR), which can be measured in serum.[7]

Synthesis of transferrin receptor and the iron storage protein ferritin are reciprocally linked to cellular iron content.[8] Untranslated regions of the mRNA for both proteins contain sequences termed the iron response element that interact with a cellular protein sensitive in its affinity to variations in cytosolic iron. Apart from the posttranscriptional regulation, transferrin receptor also appears to be transcriptionally regulated in hemoglobin synthesizing cells.[9]

The sTfR number on the cell surface reflects the iron requirement of the body. Iron deprivation has been shown to result in prompt induction of sTfR synthesis, whereas no effect is seen in anemia of chronic disease. Erythropoietic activity of bone marrow also affects the levels of sTfR; being decreased by diminished erythropoietic activity and increased when erythropoiesis is increased.[4]

The present study was done to ascertain the role of sTfR as a marker of iron deficiency anemia in the patients of CKD undergoing hemodialysis.

## Materials and Methods

### Study group

The study group consisted of 102 adult patients diagnosed to have chronic kidney disease with a GFR < 10 ml / min/1.73 sqm BSA. All patients were on maintenance hemodialysis. Patients with megaloblastic anemia were excluded.

Out of 102 patients enrolled in the study, five patients died before the time of first follow up. Out of the rest 97, 55 came for follow up at 4 weeks. One of the patients underwent renal transplantation and four patients died before the time of second follow up at six months. Out of the 50 remaining patients, 25 patients continued the treatment and came for second follow up.

These patients were on maintenance hemodialysis biweekly. They were being given weekly injections of recombinant human erythropoietin, subcutaneously as per their requirement. Intravenous iron supplementation was given using Iron (III) hydroxide polymaltose complex or iron sucrose as indicated.

The control group (C) consisted of 36 subjects. These patients were being investigated for iron deficiency anemia and had no known renal disease. sTfR levels were measured in 20 samples of apparently healthy individuals also during the study.

Anemia was treated in all subjects as per National Kidney Foundation's Kidney Disease Outcomes Quality Initiative (NKF/DOQI) which recommends starting workup for anemia when the hemoglobin is less than 11 g/dl in pre-menopausal females and pre-pubertal patients, and hemoglobin is less than 12 g/dl in adult males and post-menopausal females.[10]

Iron was estimated by ‘Ferene-S’ Method and TIBC was quantitatively estimated by Magnesium Carbonate Method on a semi-automated auto analyzer – Clima Plus using Clonital kits. Ferritin was estimated by Electrochemiluminiscene (ECLIA) immunoassay kit from Roche on the Elecsys 1010.

sTfR was quantitatively estimated by particle enhanced Immunoturbidimetric Assay kit (Orion Diagnostica) on semiautomated analyzer (Erba Chem. 5 Plus).

### Statistical analysis

Results obtained were analyzed using chi-square test, student's‘t’ test and Pearson's coefficient of correlation ‘*r*’.

## Results

The study included 102 patients and control group of 36 patients. The mean age was 48.56 ± 16.34 years in the study group and 45.91 ± 17.45 years in the control group. Diabetes mellitus was the commonest cause of CKD, with 50 patients (49.02%) suffering from it [Table 1]. The mean hemoglobin (Hb) of all patients (102) at presentation was 7.65 ± 2.1 gm% and in controls 8.5± 2.3gm%. The mean sTfR levels in apparently healthy individuals was 2.0 mg/l, and the values varied from 0.9- 2.5 mg/l. The mean sTfR level in study group (102 patients) at baseline was 3.23 ± 2.07 mg/l. The mean level of sTfR in controls was 5.16 ± 3.64 mg/l. This difference was statistically significant (*P* < 0.01) [Table 2].

**Table 1:** Distribution of patients according to etiology

Etiology	*n* (%)
Diabetes	50 (49.02)
Hypertension	9 (8.82)
CGN	14 (13.73)
Obstructive nephropathy	17 (16.67)
CIN	8 (7.84)
PKD	2 (1.96)
SLE	1 (0.98)
Nephrotic syndrome	1 (0.98)

CGN- Chronic glomerular nephritis, CIN- Chronic interstitial nephritis, PKD- Polycystic kidney disease, SLE- Systemic lupus erythrematoses

**Table 2:** Mean sTfR levels at different periods of assessment and correlation with hemoglobin

Group	Baseline		Four weeks (*n* = 55)		Six months (*n* = 25)	
	
	Mean ± SD (mg/l)		Mean ± SD (mg/l)		Mean ± SD (mg/l)	
Study group (*n* = 102)	3.23 ± 2.07		3.31 ± 1.38		2.84 ± 0.87	
Controls (*n* = 36)	5.16 ± 3.64		-		-	
*P*	Between cases and controls < 0.01		-		-	
Correlation with HB	*r* value	*P* value	*r*	*P*	*r*	*P*
	−0.028	>0.1	−0.063	>0.1	−0.274	>0.1
Study group (*n* = 55)	3.37 ± 2.43		3.31 ± 1.38		-	
*P*	Between baseline and four weeks > 0.1					
Study group (*n* = 25)	3.49 ± 2.67		3.34 ± 1.52*		2.84 ± 0.87*	
*P*	Between baseline and four weeks > 0.1				* > 0.1	

In the 55 patients who were followed up for a minimum of one month there was no statistically significant change in sTfR levels. The 25 patients, who were followed up for 6 months, also did not show any statistically significant change in sTfR levels.

On analyzing the correlation of Hb with sTfR, a negative correlation was found, though this was statistically insignificant [Table 2]. The mean value of serum iron in study group was 101.5± 9.2 *μ*g/dl at baseline and in controls was 66.65± 28.52 *μ*g/dl. This difference was statistically significant. The serum ferritin was much higher in study group (513.82 ± 505.07 ng/ml) than in the control group (88.66 ± 132.92 ng/ml). TIBC was lower (297.2 ± 100.1 *μ*g/dl) in CKD patients with a higher TSAT values (32.2 ±15.2%) as compared to the control group. The difference was statistically significant [Table 3].

**Table 3:** Mean values of other markers of anemia at baseline in cases and controls and their correlation with sTfR

Variable	Study group	Control group	*P* value	Correlation with sTfR in cases (*n*=102)	
				*r* value	*P*
Iron (μg/dl)	101.5 ± 92.32	66.65 ± 28.52	< 0.05	−0.080	> 0.10
TIBC (μg/dl)	297.2 ± 100.1	418.10 ± 103.82	< 0.001	−0.094	> 0.10
TSAT (%)	32.2 ± 15.2	17.06 ± 8.19	< 0.001	−0.084	> 0.10
Ferritin (ng/ml)	513.82 ± 505.07	88.66 ± 132.92	< 0.001	−0.075	> 0.10

The patients of CKD suffering from iron deficiency anemia may have functional or absolute iron deficiency. To differentiate between them, the patients were divided into iron replete and iron deplete groups based on TSAT<20% and ferritin < 100 ng/ml.

Seventy-three (71.57%) patients had sufficient iron stores as per the guidelines of NKF / DOQI. Twenty-nine (28.43%) patients had TSAT < 20% or ferritin < 100 ng/ml or both indicating depletion of iron stores. Three patients had both TSAT < 20% and ferritin < 100 ng/ml indicating absolute iron deficiency. There was a statistically significant difference in level of sTfR levels in these groups (*P* <0.05) [Table 4].

**Table 4:** Comparison of sTfR levels in patients on dividing into iron depleted (TSAT<20% and Ferritin<100ng/ml) and iron sufficient group (TSAT>20% and Ferritin >100ng/ml) as per the guidelines of NKF / DOQI

	Iron replete group	Iron deplete group
*n*	73 (71.57%)	29 (28.43%)
Mean sTfR	3.39 ± 2.36 mg/L	2.82 ± 0.98 mg/L

*P* < 0.05

When sTfR values were correlated with iron, TIBC, TSAT and ferritin in patients with CKD and controls, no statistically significant correlations were found. The change in values of these parameters on follow up also did not show statistically significant correlation with sTfR [Table 3].

Many authors have used sTfR/ log ferritin ratio, sTfR F index for their analysis and given a cutoff value to diagnose iron deficiency anemia. In our study the mean of sTfR F index in cases was 1.3 (range 0.3-6.6) and in controls was 4.5 (range 1.5-15.5). It can be inferred that a ratio of >1.4 indicates co existence of iron deficiency anemia with anemia of chronic disease while ratio of up to 1.3 indicates anemia of chronic disease only.

## Discussion

A total of 102 patients were enrolled for the study, nine patients died and one underwent renal transplantation before the completion of the study. Fifty five patients could be followed- up for 4 weeks, while only 25 patients completed the study and were followed up for the complete 6 months.

The main cause of CKD in the present study was diabetes mellitus (49.02%). Agarwal in 2005 observed that in India, diabetes and hypertension were responsible for 40-50% of all cases of CKD.[1]

The prevalence of anemia in the patients was 96.08% and the mean hemoglobin at baseline was 7.65±2.1 g%. Talwar *et al*. in 2002 reported a prevalence of 94%[11] while Maiz *et al*.[12] reported 87.8% of their patients had anemia in their study. Prevalence of iron deficiency anemia in CKD patients has been reported as high as 60% in the study by Talwar *et al*.[11] The mean hemoglobin reported by Singh *et al*. in 1999 in Indians was 7.27 ± 1.26 g%,[13] while Braun *et al*.[14] observed hemoglobin levels of 10.5 ± 1.4 g% and Fusaro *et al*.[15] of 11.4 ± 1.2 g% in their respective studies.

On comparing the sTfR levels in controls and patients with CKD, a statistically significant difference was found, with a *P* value of < 0.01. The mean sTfR level in patients at enrolment was 3.23 ± 2.07 mg/l. The mean level of sTfR in controls was 5.16 ± 3.64 mg/l. There was no data available from India regarding the levels of sTfR in CKD patients. Being an expensive test, no work has been reported from India, the values observed by researchers in other countries were understandably different and not comparable. Fusaro *et al*.[15] observed sTfR levels of 1.94 ± 0.83 mg/l while Tarng and Huang.[16] had reported values ranging from 1.246 – 1.573 mg/l. A mean sTfR of 2.3 ± 1.4 mg/l was observed by Fernandez- Rodriguez *et al*.[17]

On follow up of these hemodialysis-dependent CKD patients for six months, who were also receiving intravenous iron and erythropoietin, no statistically significant change in the levels of sTfR was found. Also, there was no statistically significant difference in hemoglobin levels though some improvement was seen, from 7.65±2.1 gm/dL at enrolment to 8.51± 1.88 gm/dL at 6 months. Thus, no statistically significant change in Hb and sTfR levels was observed, which is difficult to explain.

On studying the correlation of hemoglobin with sTfR, a negative correlation was found (r = − 0.028) which was statistically insignificant. Tonbul *et al*. in 1998 reported an inverse correlation between hemoglobin and sTfR values in hemodialysis patients on recombinant erythropoietin treatment.[18] Chiang *et al*. in 2002 observed that concentration of sTfR positively correlated with hematocrit and hemoglobin.[19] Lorenzo *et al*. in 2001 also reported a positive correlation between hemoglobin, hematocrit and sTfR.[20] Further studies need to be done to have a consensus on the correlation of hemoglobin with sTfR in hemodialysis patients on iron and erythropoietin therapy.

The mean values of serum ferritin, TIBC and TSAT showed statistically significant difference between cases and controls. The control group presented with the biochemical profile diagnostic of iron deficiency anemia with low ferritin and TSAT. However, in the case of the study group, ferritin levels were markedly high (515.82± 505.07 ng/ml) as compared to (88.66 ± 132.92 ng/ml) the control group. TIBC values were lower (297.2 ± 100.1*μ*g/dl) in study group as compared to 418.10 ± 103.82*μ*g/dl in controls. These results emphasize the limitation of these parameters to diagnose iron deficiency anemia in CRF patients as ferritin is also an acute phase reactant and TIBC a negative acute phase reactant. Uremia per se has been described as a chronic inflammatory state and these parameters can not represent the true state of iron status in these patients.[21]

The levels of sTfR were lower (2.82 ± 0.98 mg /l) in iron deplete group as compared to the iron replete group(3.39 ± 2.36 mg/l) indicating that sTfR is not a good marker of IDA in CKD. Raised sTfR levels in iron replete group may be due to ongoing erythropoiesis in these patients.

In the present study no significant correlation of sTfR with iron, TIBC, ferritin or transferrin saturation was found in hemodialysis patients on follow up. Tarng and Huang in 2002 reported a significant correlation of sTfR with ferritin and transferrin saturation.[16] However, in the study done by Fusaro *et al*. in 2005, no correlation was found between sTfR and TSAT or between sTfR and ferritin.[15]

The role of sTfR as a marker of iron deficiency anemia in CRF patients remains doubtful. This is due to complexity of iron metabolism in these patients. They may have absolute iron deficiency as well as functional iron deficiency due to erythropoietin therapy and inadequate iron supplementation. Further, levels of sTfR can be raised in both iron deficiency and erythropoiesis.
